# Deep sequencing approach for investigating infectious agents causing fever

**DOI:** 10.1007/s10096-016-2644-6

**Published:** 2016-05-14

**Authors:** T. N. Susilawati, A. R. Jex, C. Cantacessi, M. Pearson, S. Navarro, A. Susianto, A. C. Loukas, W. J. H. McBride

**Affiliations:** College of Medicine and Dentistry, James Cook University, PO Box 902, Cairns Hospital, Cairns, QLD Australia; Department of Microbiology, Faculty of Medicine, Sebelas Maret University, Jl. Ir. Sutami 36 A, Surakarta, Central Java Indonesia; Population of Health and Immunity Division, Walter and Eliza Hall Institute, Parkville, VIC Australia; Faculty of Veterinary and Agricultural Sciences, The University of Melbourne, Parkville, VIC Australia; Australian Institute of Tropical Health and Medicine, James Cook University, Cairns, QLD Australia; Department of Veterinary Medicine, University of Cambridge, Cambridge, UK

## Abstract

Acute undifferentiated fever (AUF) poses a diagnostic challenge due to the variety of possible aetiologies. While the majority of AUFs resolve spontaneously, some cases become prolonged and cause significant morbidity and mortality, necessitating improved diagnostic methods. This study evaluated the utility of deep sequencing in fever investigation. DNA and RNA were isolated from plasma/sera of AUF cases being investigated at Cairns Hospital in northern Australia, including eight control samples from patients with a confirmed diagnosis. Following isolation, DNA and RNA were bulk amplified and RNA was reverse transcribed to cDNA. The resulting DNA and cDNA amplicons were subjected to deep sequencing on an Illumina HiSeq 2000 platform. Bioinformatics analysis was performed using the program Kraken and the CLC assembly-alignment pipeline. The results were compared with the outcomes of clinical tests. We generated between 4 and 20 million reads per sample. The results of Kraken and CLC analyses concurred with diagnoses obtained by other means in 87.5 % (7/8) and 25 % (2/8) of control samples, respectively. Some plausible causes of fever were identified in ten patients who remained undiagnosed following routine hospital investigations, including *Escherichia coli* bacteraemia and scrub typhus that eluded conventional tests. *Achromobacter xylosoxidans*, *Alteromonas macleodii* and Enterobacteria phage were prevalent in all samples. A deep sequencing approach of patient plasma/serum samples led to the identification of aetiological agents putatively implicated in AUFs and enabled the study of microbial diversity in human blood. The application of this approach in hospital practice is currently limited by sequencing input requirements and complicated data analysis.

## Introduction

Acute undifferentiated fever (AUF) is caused by a variety of causes, producing a range of clinical manifestations with acute fever as a unifying symptom. Most clinicians and researchers define acute fever as evidence of raised body temperature to ≥38 °C for ≤3 weeks, without detection of systemic disease or the focus of infection or inflammation after initial clinical evaluation and basic laboratory investigations [1]. This condition poses a diagnostic challenge for clinicians due to non-specific clinical features and the indistinctive profile of routine blood tests. Without a specific aetiological diagnosis, the treatment of AUF is often based on an ‘educated guess’ or a syndromic approach that often leads to inappropriate treatment [2, 3].

It has long been known that infection is the main cause of fever, particularly in the acute stage. Unfortunately, there are hundreds of possible aetiologies of fever, such that conventional diagnostic tools are often either unavailable or restricted to a subset of the ‘most likely’ infectious agents due to the high costs associated with laboratory testing. The limitation of the current diagnostic approach causes a significant proportion of fever to go undiagnosed. Indeed, the frequencies of undiagnosed AUFs in Asian tropical countries ranges from 8 to 80 % [1].

The wide availability of nucleic acid [i.e. polymerase chain reaction (PCR)-based] assays in clinical laboratories provides sensitive and specific detection of pathogens. However, while techniques such as multiplex PCR can provide simultaneous detection of multiple pathogens, this approach is impractical for more than a handful of pathogens in any one assay [4, 5] and is not capable of detecting novel pathogens [6]. Diagnostic microarrays can expand detection capacity considerably, allowing simultaneous detection of tens of pathogens or more [7, 8], but these too are limited for the detection of novel or emerging pathogens [9].

The advent of next-generation sequencing (NGS) [10] provides a basis for unbiased identification of infectious agents associated with AUF, as well as the capacity to identify novel and emerging pathogens [9, 11, 12]. This study aimed to evaluate the practical use of NGS technology as a diagnostic tool for the identification of infectious agents causing fever. This method is referred to as metagenomic deep sequencing and has been used in previous studies to determine the agents responsible for dengue-like illnesses [9] and acute haemorrhagic fever [13].

## Materials and methods

### Sample collection

**Fig. 1:**
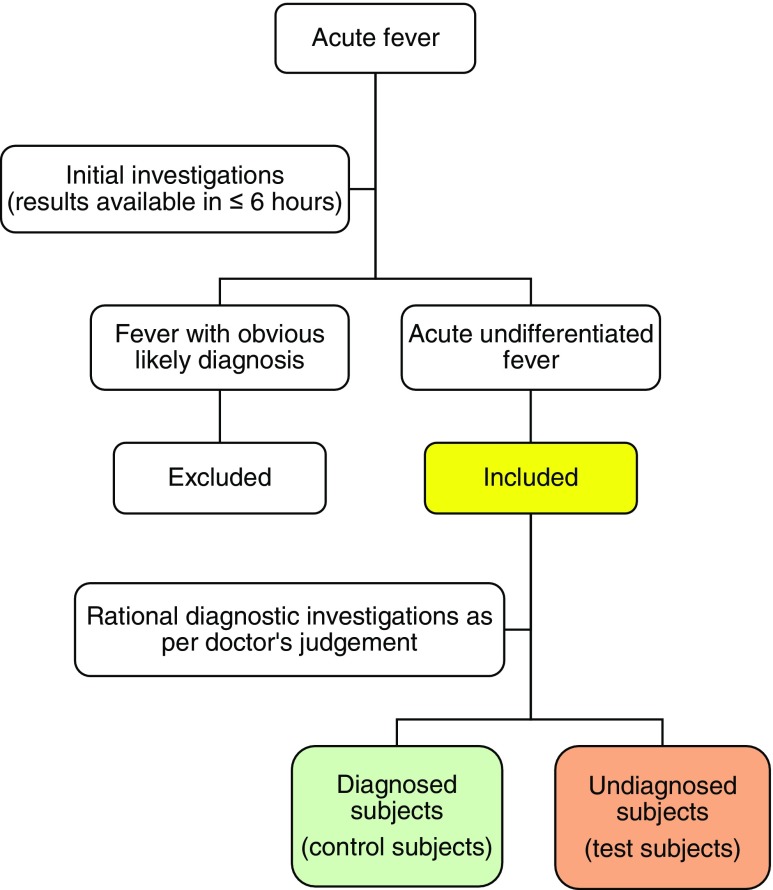
Flow chart of patient selection. Definitions: Acute fever is an increase of body temperature to 38 °C or more for a period of 21 days or less. Initial investigations refer to comprehensive clinical assessment and basic laboratory and radiology tests; this included tests that are normally reported within 6 h from admission. Comprehensive clinical assessment includes complete history taking and thorough physical examination. Basic laboratory tests usually include complete blood count and urinalysis. Basic radiology tests could include chest X-ray, abdomen and pelvic X-ray, and ultrasonography. Fever with obvious likely diagnosis is any case of fever with definitive diagnosis immediately after initial investigations. This includes fever cases with an obvious focus of infection or local inflammation, such as community-acquired pneumonia, urinary tract infection, skin and soft tissue infection, bone and dental infection, pelvic inflammatory disease and intra-abdominal infection. Acute undifferentiated fever is any case of acute fever with unclear aetiology and the results of initial investigations are not conclusive in achieving a diagnosis. Thus, the condition is characterised by a requirement for further investigation to explain the cause of fever and to consider differential diagnoses. Rational diagnostic investigations are further tests as judged by an attending doctor to determine the cause of fever, such as further serology, cerebrospinal fluid analysis and/or advanced radiology tests [computed tomography (CT) scan, magnetic resonance imaging (MRI)]. Samples were collected from both groups of participants (diagnosed subjects and undiagnosed subjects) for fever investigation using the deep sequencing approach

We collected 40 plasma/serum samples from patients who presented to Cairns Hospital, a tertiary hospital in Cairns, Far North Queensland, Australia (16.9256° S, 145.7753° E). The inclusion criteria included patients aged 16 to 65 years who had raised temperature of ≥38 °C or history of fever with feeling cold or shivering for up to 21 days with no evident focus of infection and no obvious cause of fever after initial clinical, radiology and laboratory evaluation; this included tests for which results were normally reported within 6 h from admission. Thus, a specific diagnosis at the time of patient recruitment was unavailable. Subsequent investigation(s) determined by the attending doctors ascertained an aetiological diagnosis in a subset of study participants (control subjects), while other participants (test subjects) remained undiagnosed (Fig. 1).

### Sample preparation and sequencing

DNA was isolated from 200 μl of plasma/serum using the QIAamp® DNA Mini Kit (Qiagen), while RNA was isolated from 250 μl of sample mixed with 750 μl of TRIzol® LS reagent (Life Technologies), as per the manufacturer’s protocol. Each sample preparation was performed in duplicate. Following RNA isolation, genomic DNA was removed from RNA samples using DNase I, Amplification Grade (Sigma Aldrich). Amplification of DNA and RNA was conducted according to the SeqPlex Enhanced DNA Amplification Kit and the SeqPlex RNA Amplification Kit (Sigma Aldrich) protocols, resulting in double-stranded DNA and cDNA amplicons, respectively. The GenElute PCR Clean-Up Kit (Sigma-Aldrich) was used for the purification of products from the SeqPlex DNA and RNA amplification kits. The quantity, size and purity of DNA/cDNA amplicons were determined using gel electrophoresis (1.5 % agarose) and a NanoDrop 2000 spectrophotometer (Thermo Scientific), as requested by the commercial sequencing service used for the study: the Australian Genome Research Facility (AGRF). The samples that passed AGRF quality assessment were processed into library preparations using the TruSeq Nano DNA Library Preparation Kit protocol (Illumina). Paired-end (PE) 100-bp sequencing was conducted using an Illumina HiSeq 2000 instrument.

### Bioinformatics analysis

**Fig. 2:**
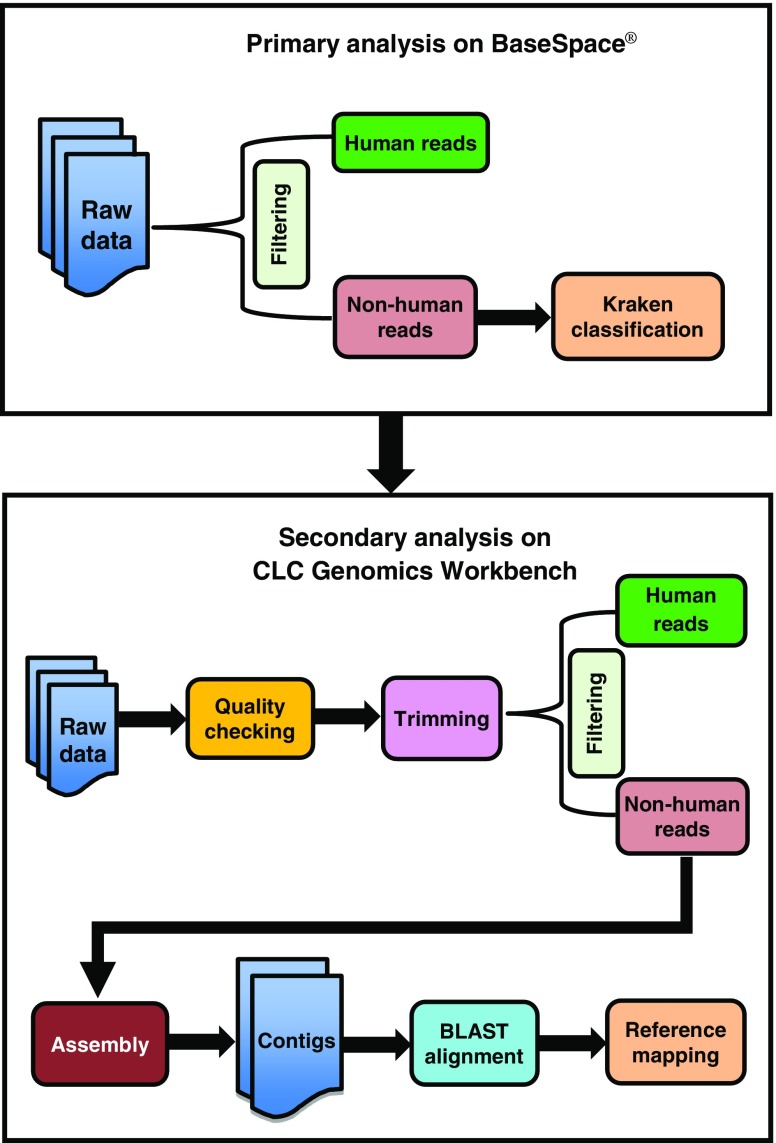
Analysis workflow. Primary and secondary analyses were performed sequentially for all samples and performed in parallel for the purpose of validation. Sequential analysis means that all reads were uploaded to the BaseSpace® server, followed by analysis of non-human reads not classified by Kraken in the CLC server. Validation of next-generation sequencing (NGS) analysis was performed on positive control samples only by analysing all reads using the BaseSpace® and CLC servers in parallel

Identification of pathogens associated with AUF was performed on two cloud computing servers: BaseSpace® (Illumina) and CLC Genomics Workbench (Qiagen) (Fig. 2). First, the raw sequence data obtained from AGRF were uploaded onto the BaseSpace® server; then, human sequences in each dataset were identified by alignment to the human reference genome (hg19) using the program SNAP version 1.0beta.14 [14] and removed. The remaining sequences were then classified using the default parameters of the program Kraken version 0.10.4-beta [15], based on their homology with organisms in the MiniKraken 20140330 database, which contains a collection of complete bacterial, archaeal and viral genomes available from the National Center for Biotechnology Information (NCBI) RefSeq database.

Any non-human reads not classified by Kraken were imported to the CLC Genomics Workbench server. The quality of the sequence data was examined using the FastQC tool [16]. The reads were then processed using the CLC Trim Sequences tool to remove adapters, low quality bases, ambiguous nucleotides, terminal nucleotides (25–35 nucleotides from the 5’ end) and short sequences (less than 24 nucleotides). Then, reads were mapped and filtered a second time using the CLC read mapper program to the human reference genome (hg19). All remaining reads were assembled using the CLC De Novo Assembly tool. Assembled contigs were compared with the NCBI non-redundant database using the Basic Local Alignment Search Tool (BLAST) [17]. The BLASTn program optimised for highly similar sequences (megablast) was used to search nucleotide databases for sequence(s) that matched a nucleotide query. The nearest matching sequence (e-value threshold ≤10^−5^) was accepted as the most likely homolog for each contig. As an independent assessment of Kraken, we also ran all reads from the control samples through the CLC workflow without prior SNAP filtering/Kraken analysis.

The results of primary (Kraken) and secondary (CLC) analyses were screened for pathogens known to cause prominent symptoms in infected patients. Following this, the probable causative agents were listed according to the number of reads (from largest to smallest) obtained from the primary analysis. The BLAST e-value was reported if the pathogen was detected in the secondary analysis as well. When the sequencing was performed on duplicate samples, only the results from the sample that produced the largest sequencing dataset were reported. Finally, the results of bioinformatics analysis in conjunction with supporting clinical data and laboratory findings were used to inform diagnosis.

## Results

**Fig. 3:**
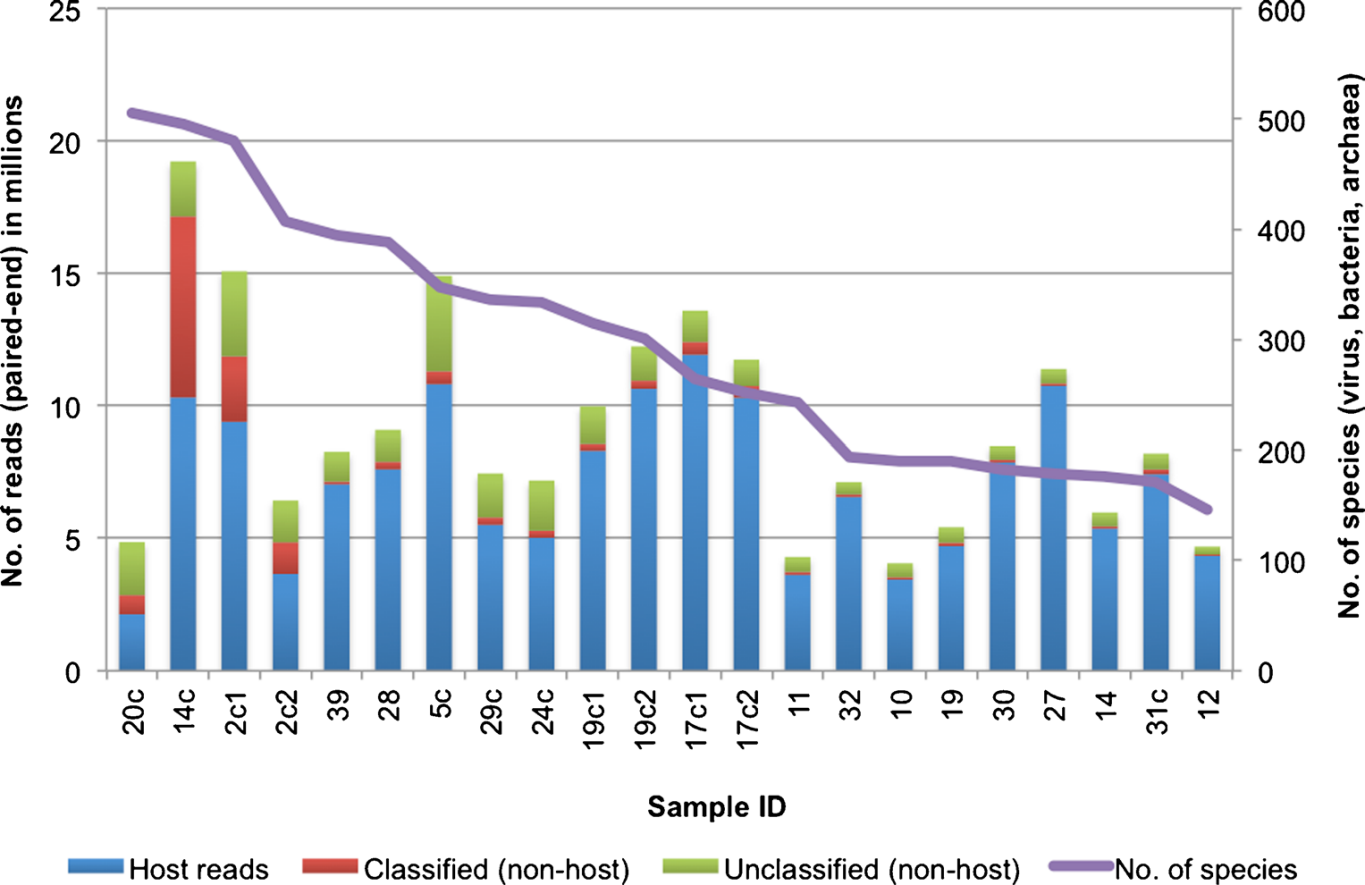
Summary of Kraken analysis on reads generated by Illumina HiSeq 2000. cDNA samples from patient ID# 002, 017 and 019 were prepared and sequenced in duplicate (sample IDs 2c1, 2c2, 17c1, 17c2, 19c1 and 19c2)

Of the 40 DNA and cDNA samples that we prepared in duplicate, only 22 samples from 17 participants (eight samples from seven control subjects and 14 samples from ten test subjects) met the quantity and quality requirements for deep sequencing. From these, between 4 and 20 million reads per sample were generated. The majority (43.67–94.38 %) of these reads were of human origin, with only a small proportion of non-human reads being classified by Kraken. The number of viral, bacterial and archaeal species reported by Kraken varied considerably, from 146 to 505 species per sample (Fig. 3).

**Fig. 4:**
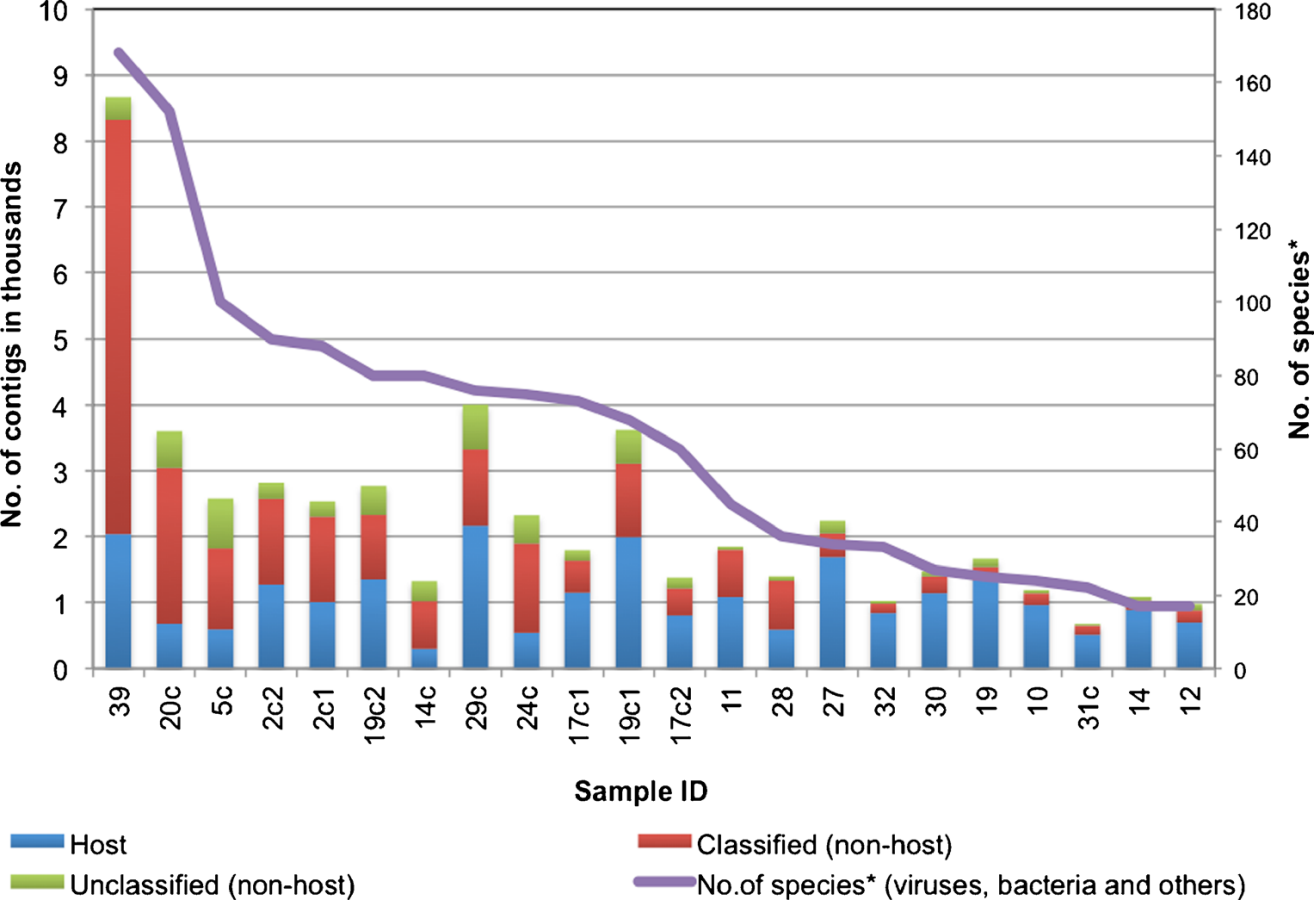
Summary of CLC Genomics analysis on Kraken-unclassified reads *BLASTn optimised for highly similar sequences (megablast) was performed to search the homology between query sequences and reference sequences in the database. If multiple significant similarities matched with a single species, only the highest scoring hit was reported. A similarity was considered significant at e-value ≤10^−5^

The secondary analysis facilitated further classification of reads left unclassified by Kraken. Analysis with the CLC Genomics Workbench revealed that Kraken-unclassified reads still contained human sequences, accounting for 18.9–81.7 % of the total contigs in each sample. Non-host contigs classified by BLAST analysis included viruses, bacteria and other organisms, such as archaea (e.g. *Sulfolobus* sp.), fungi (e.g. *Saccharomyces* sp., *Cryptococcus* sp., *Penicillium* sp.), algae (e.g. *Navicula gregaria*), plants (e.g. rice, tomato, grain, tobacco), protozoa (e.g. *Toxoplasma gondii*, *Plasmodium berghei*), human parasites (e.g. roundworm, tapeworm, pinworm) and larger animals (e.g. snail, fish, rat, monkey, orangutan, gorilla). Following analysis, 2.7–29.5 % of the total contigs in each sample remained unclassified (Fig. 4).

**Fig. 5:**
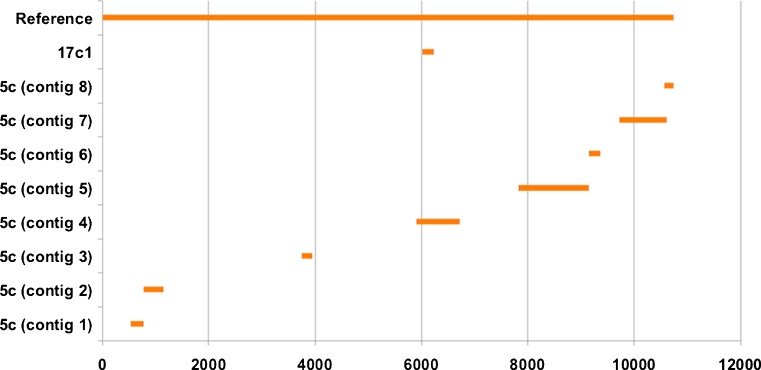
Mapping of dengue virus 1 contigs against dengue virus 1 complete genome of 10,735 bp genomic DNA (NCBI Reference Sequence: NC_001477.1). The X-axis represents the size and position of the genome/contigs in base pair (bp); the Y-axis represents the dengue virus 1 reference genome and the dengue virus 1 contig(s) found in the sample ID# 5c and 17c1; both are cDNA samples from patient ID# 005 and 017, respectively

Table 1 compares the outcomes of hospital investigations and NGS analyses. Confirmation of diagnosis by NGS means that at least one read or one contig was assigned to the infectious organism. Kraken analysis confirmed the specific diagnoses obtained by other means in 7/8 (87.5 %) of control samples, whereas CLC analysis only concurred with the results of clinical tests in 2/8 (25 %) of control samples, both of whom had dengue virus 1 infection: patient ID# 005 and 017. The CLC reference mapping aligned the position of the virus contigs with the dengue virus 1 reference genome (accession number: NC_001477.1). In the first dengue case (ID# 005), the CLC analysis constructed eight contigs of dengue virus 1 with length 166 to 1328 bp, whereas in the second dengue case (ID# 017), only one contig of dengue virus 1 was available, with a length of 217 bp (Fig. 5).

**Table 1:** Validation of diagnosis in eight samples originating from seven control subjects

Patient (sample) ID	Diagnosis	Results from conventional hospital diagnostic approach	Time of sample collection for NGS study	Detection of pathogen in NGS analysis	
				Kraken – no. of reads	CLC – no. of contigs
005 (5c)^a^	Dengue	PCR+, NS1+, IgM−	Fever day-4	Yes – 105,738	Yes – 8
010 (10)^b^	Leptospirosis	IgM+	Fever day-9, antibiotics day-3	Yes – 1	No
017 (17c1)^a^	Dengue	NS1+, IgM+	Fever day-8	Yes – 64	Yes – 1
017 (17c2)^a^	Dengue	NS1+, IgM+	Fever day-8	Yes – 4	No
020 (20c)^a^	Dengue	NS1+, IgM+	Fever day-6	Yes – 25	No
024 (24c)^a^	Measles	PCR+, IgM+	Fever day-4	No	No
031 (31c)^a^	Dengue	PCR−, NS1+, IgM+	Fever day-8	Yes – 6	No
032 (32)^b^	*S. pyogenes* bacteraemia	Blood culture+	Fever day-2, antibiotics day-1	Yes – 1	No

^a^Sequencing was performed on cDNA samples

^b^Sequencing was performed on DNA samples

Our deep sequencing approach identified some plausible causes of fever in 80 % (8/10) of the test subjects (ID# 002, 011, 014, 019, 027, 029, 030, 039) who remained undiagnosed after routine hospital investiagations (Table 2). In particular, our analyses confirmed the aetiological diagnosis of two AUF cases that eluded conventional investigation methods. A high number of *Escherichia coli* sequences were detected in the sample of a fatal diarrhoea case with a sterile blood culture (patient ID# 002) and *Orientia tsutsugamushi* was detected in a PCR-negative patient with clinical features of scrub typhus (patient ID# 011). Furthermore, the results of deep sequencing highlighted a surprising microbial diversity in a ‘sterile’ environment; that is, human blood. Table 3 shows 61 organisms that were present in all samples and their relative abundance.

**Table 2:** Plausible next-generation sequencing (NGS) diagnoses in patients with undiagnosed fevers

Patient (sample) ID	Clinical data	Pathology and radiology findings from conventional investigation at Cairns Hospital	Plausible NGS diagnosis
002 (2c1, 2c2)^a^	A 59-year-old indigenous man with diarrhoea. Other active problems: ischaemic heart disease, pulmonary hypertension, atrial flutter, type 2 diabetes mellitus, hypertension and dyslipidaemia. During admission, the patient commenced gentle rehydration, ceftriaxone and doxycycline. Ongoing diarrhoea, dehydration and low blood pressure led to patient transfer to intensive care on day 4. He was given poor prognosis and transferred back to the ward for comfort measures. He died on day 18 due to multi-organ failure.	• Serum urea: 51.7 mmol/l (↑), creatinine: 1000 μmol/l (↑), ALT: 414 U/l (↑), AST: 1290 U/l (↑), CRP: 102 mg/l (↑) • Platelet: 60 × 10^9^/l (↓), WBC: 23.2 × 10^9^/l (↑), with neutrophil predominant (20.69 × 10^9^/l) • Antinuclear antibody (ANA): (+) • Blood culture: (−) • Dengue and hepatitis C serology: (−) • Faecal microbiology: normal faecal flora was absent, *Clostridium difficile* screen (−), no evidence of parasitic infection on microscopic examination, and no *Salmonella*, *Shigella*, *Yersinia* or *Campylobacter* grown on culture. • Chest X-ray: cardiomegaly, no consolidation in lungs; chest CT: small pleural effusion; abdomen CT: ascites, fatty liver, hepatomegaly and splenomegaly • Ascites analysis (chemistry and microbiology): normal	*Escherichia coli* (57,657 reads, e-value 4.3E-49).
011 (11)^b^	A 40-year-old man with a 3-day history of rash, fever, myalgia and nausea, following recent travel to the Torres Strait Islands and possible contact with mites. Erythematous blanching papules rash were found on trunk, chest and extremities, and there was an eschar on his upper left arm. He showed marked clinical improvement with doxycycline.	• Serum ALT: 177 U/l (↑), AST: 164 U/l (↑), CRP: 97 mg/l (↑). • Platelet: 108 × 10^9^/l (↓), WBC: 3.4 × 10^9^/l (↓). • Urinalysis: no abnormality detected • Blood culture: (−) • Malaria screening: (−) • Flavivirus, Q fever and *Leptospira* serology: (−) • Spotted fever group (SFG) *Rickettsia* and scrub typhus PCR: (−)	*Orientia tsutsugamushi* (11 reads).
012 (12)^b^	A 31-year-old female from the Atherton Tablelands presenting with abdominal pain, nausea, vomiting and constipation on a background of inflammatory bowel syndrome. The patient was given analgesia and was advised to return to the emergency department if the pain returned.	• Serum ALT: 545 U/l (↑), AST: 428 U/l (↑) • ANA: (+) • Blood culture: (−) • Hepatitis B and C serology: (−) • Serology and PCR for CMV, *Leptospira* and Q fever: (−) • Chest X-ray: no abnormality detected • Abdomen CT: splenomegaly	Nil significant.
014 (14, 14c)^a,b^	A 55-year-old man from Babinda with fever, myalgia and blanching maculopapular rash with history of mosquito bites 2 weeks prior to presentation. The patient’s wife (ID# 019) concomitantly presented with a more severe variant of the same illness. He clinically improved with ceftriaxone and doxycycline.	• Serum ALT: 63 U/l (↑), AST: 56 U/l (↑) • Platelet: 122 × 10^9^/l (↓) • Blood culture: (−) • Serology for EBV, *R. rickettsia*, *O. tsutsugamushi*, *Mycoplasma pneumoniae*, *Leptospira*, flavivirus: (−) • PCR for *Rickettsia* (SFG and typhus group), *Streptococcus pneumoniae*, *Neisseria meningitidis* and *Leptospira*: (−) • *S. pneumoniae* and *Legionella* antigens in urine: (−)	Dengue virus (25 reads).
019 (19, 19c1, 19c2)^a,b^	A 57-year-old female transferred from Babinda hospital and was the spouse of patient ID# 014. She had an 8-day history of fever, rash, retro-orbital headache, myalgia, malaise, vomiting and diarrhoea. The patient was hypotensive at initial presentation to Babinda hospital and was given ceftriaxone, vancomycin and doxycycline intravenously prior to transfer. The patient had evidence of multi-organ failure at transfer to Cairns Hospital and was intubated. She died with a diagnosis of septic shock.	• Serum urea: 43.7 mmol/l (↑), creatinine: 839 μmol/l (↑), ALT: 1170 U/l (↑), AST: 6160 U/l (↑) • Platelet: 14 × 10^9^/l (↓), WBC: 35.1 × 10^9^/l (↑) with neutrophil predominance (30.57 × 10^9^/l) • Blood culture: (−) • Serology for *Leptospira*, *S. pneumoniae*, EBV, CMV, flavivirus, Q fever, *R. typhi*, *R. rickettsii*, *O. tsutsugamushi*, SFG and typhus group *Rickettsia*: (−) • PCR for *Leptospira*, dengue, scrub typhus, *S. pneumoniae* and *N. meningitidis*: (−) • Malaria screen: (−)	1. EBV (7023 reads). 2. Dengue virus (19 reads). 3. *R. conorii* (1 read). 4. *A. baumannii* (2514 reads, e-value 2.0E-68). 5. *Listeria monocytogenes* (13 reads).
027 (27)^b^	A 29-year-old man presented with fever, generalised weakness, nausea, vomiting, diarrhoea, dry cough and headaches after returning from Thailand, where he had spent some time in the jungle. The provisional diagnosis was likely arbovirus infection. He was given doxycycline to cover leptospirosis and clinically improved.	• Serum urea: 11.1 mmol/l (↑), creatinine: 181 μmol/l (↑), ALT: 94 U/l (↑), AST: 68 U/l (↑), CRP: 487 mg/l (↑) • Platelet: 90 × 10^9^/l (↓), WBC: 15.3 × 10^9^/l (↑) • Blood culture: (−) • Faeces culture: no *Salmonella*, *Shigella*, *Yersinia*, *Campylobacter* • Malaria screening: (−) • *Leptospira* and dengue serology: (−) • Respiratory viruses PCR: (−)	1. Dengue virus (11 reads). 2. *Salmonella enterica* (3 reads).
028 (28)^b^	A 64-year-old man with 4-day fever, chills, sore eyes and ulcerated mouth. He had a tick bite recently and had been on a cruise from Vanuatu through the Solomon Islands to Papua New Guinea. He was taking several medications for high blood pressure and type 2 diabetes mellitus.	• Serum ALT: 64 U/l (↑), AST: 43 U/l (↑) • WBC: 0.8 × 10^9^/l (↓), neutrophils 0.02 × 10^9^/l (↓), platelet: 147 × 10^9^/l (↓) • Screening for malaria, hepatitis B, hepatitis C and HIV: (−) • Serology for CMV, EBV, *R. rickettsii*, *Leptospira*, flavivirus, Q fever, *Brucella*, *Cryptococcus* and *M. pneumoniae*: (−) • Serology for *O. tsutsugamushi*: weakly positive (total immunoglobulin: 128) • PCR for HSV 1 and 2: (−) • Chest X-ray: clear • Blood and urine cultures: (−)	The patient’s medical notes were reviewed and it was found that the patient had prolonged neutropaenia (up to 4 weeks). It is likely that the patient had drug-induced febrile neutropaenia and the results of NGS analysis were not considered.
029 (29c)^a^	A 38-year-old female admitted with possible measles. Initially, the patient had back pain and fevers without urinary symptoms. She was started on antibiotics for suspected urinary tract infection. Subsequently, she developed a rash, which began on her face and spread to her torso and limbs. She worked as a housekeeper and had no exposure to potential allergens or new chemicals. The patient was admitted with strict respiratory isolation. On day 1 of admission, the doctor suspected a small bite on the patient’s back. This lesion was reviewed by the infectious diseases team and the impression was rickettsial in nature. Fevers were improving with doxycycline.	• Serum ALT: 448 U/l (↑), AST: 519 U/l (↑) • Platelet: 99 × 10^9^/l (↓) • Serology for CMV, EBV, dengue, Ross River, alphavirus, Barmah Forest, Sindbis, Chikungunya, Q fever, *Leptospira*, *Brucella*, *R. rickettsii*, *O. tsutsugamushi*, rubella and measles: (−) • Measles PCR: (−)	1. Jingmen tick virus (52 reads). 2. *R. africae* (10 reads).
030 (30)^b^	An 18-year-old man with fever, chills, headache, muscle pain, joint pain, back pain, cough, sore throat, nausea and rash. He had history of a tick bite on the upper right thigh when camping at Tinaroo Dam, Atherton Tablelands. He had been taking doxycycline for approximately 2 days, as prescribed by a 24-hour medical centre. The patient continued on doxycycline for 2 weeks.	• CRP: 37 mg/l (↑) • Platelet: 125 × 10^9^/l (↓) • *R. rickettsii* and *O. tsutsugamushi* serology: (−)	1. Jingmen tick virus (32 reads). 2. *R. africae* (1 read).
039 (39)^b^	A 57-year-old man with gradual onset of malaise, vomiting, myalgia, fevers and white productive cough. The provisional diagnosis was viral infection with possible acute renal failure secondary to viral illness. He was treated with doxycycline and Augmentin.	• Serum creatinine: 157 U/l (↑), AST: 40 U/l (↑) • Chest X-ray was normal • *Leptospira* and flavivirus serology: (−)	Dengue virus (27 reads).

^a^Sequencing was performed on cDNA samples

^b^Sequencing was performed on DNA samples

**Table 3:**
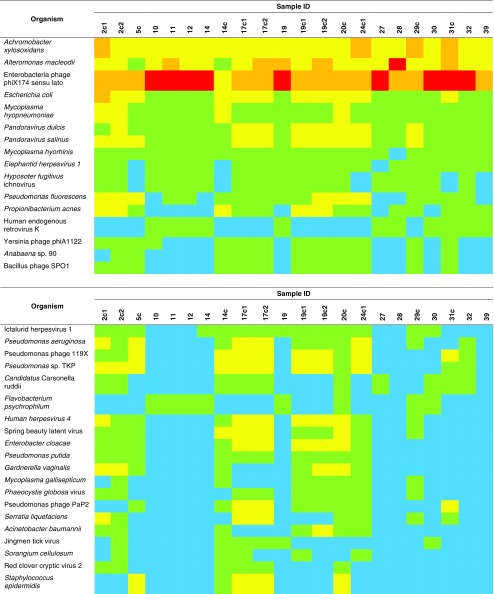

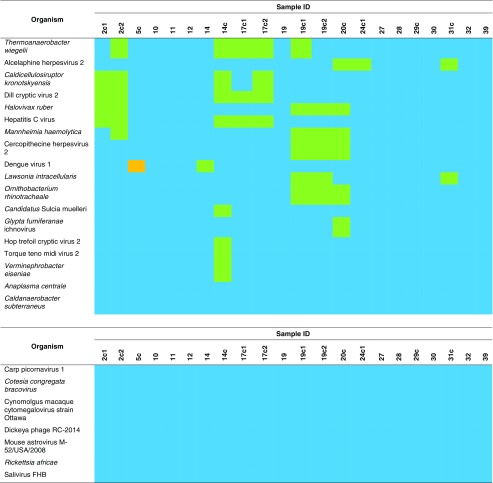
Organisms that present in all samples, detected by Kraken analysis

Relative abundance (proportion of organism reads among non-host reads) is shown by graded colour: “blue” indicates <0.01 %, “green” indicates 0.01 % - <0.1 %, “yellow” indicates 0.1 % - <1 %, “orange” indicates 1 % - <10 % and “red” indicates 10–18.48 %

## Discussion

To date, there has been little research undertaken to investigate the application of NGS in fever investigation, possibly due to the relatively high costs and complex methods of sample preparation and data analysis. Since the costs associated with NGS are proportional to the amount of sequence data generated per sequencing run, one way to reduce the sequencing costs is by requesting the minimum amount of sequence data without compromising the sensitivity of detection of the pathogen. This can be achieved, for instance, by reducing human DNA contamination to maximise the yield of pathogen sequences. Most human DNA is cellular in origin; thus, cell-free samples such as plasma and serum are expected to have a higher ratio of pathogen/human DNA than whole blood.

Previous studies have used deep sequencing to facilitate the discovery of novel viruses in plasma/serum samples [13, 18–21], but the use of such samples in the present study posed a challenge. Despite achieving low-level human DNA contamination, which is necessary to keep sequencing costs minimal, the plasma and serum samples contained very low quantity and quality of nucleic acids. Commercial sequencing services that we contacted required at least 100 ng of DNA/RNA per sample, which is challenging to achieve from plasma/serum volumes typically collected for routine blood diagnosis. The attempt to meet the minimum input requirement for sequencing necessitated amplification, which could introduce biases during sequencing [22]. Furthermore, every additional step carried out during sample preparation is a potential source of contamination, and might cause further degradation of the nucleic acids.

While the primary aim of this study was to evaluate the use of NGS in detecting pathogens associated with AUFs, our data provide an insight into the microbial diversity in human blood (Table 3). We presumed that the origins of these microorganisms are either from experimental reagents (i.e. *Achromobacter xylosoxidans*, *Alteromonas macleodii*, enterobacteria phage), cross-contamination from one sample to another (i.e. hepatitis C virus, dengue virus), from the skin during phlebotomy (i.e. *Propionibacterium acnes*, *Staphylococcus epidermidis*) or from the blood itself [i.e. torque teno midi virus (TTMDV), human herpesvirus 4].

*Achromobacter xylosoxidans* can be found in water environments and has been isolated from both immunocompetent and immunocompromised patients with bacteraemia, chronic otitis media, meningitis, urinary tract infections, abscesses, osteomyelitis, corneal ulcers, prosthetic valve endocarditis, peritonitis and pneumonia [23, 24]. The bacteria are not a typical component of human flora and have low virulence [25]. Infection with *A. xylosoxidans* is widely considered to be opportunistic, and the source of infection is usually found to be a contaminated solution [23]. The bacteria can survive in aqueous environments with minimal nutrients, so it is likely that the relatively high abundance of these bacteria (0.16–1.38 % of non-host reads) indicates contamination from the water or reagent used during sample preparation. It has been reported previously [26] that sequence-based microbiome analyses is susceptible to DNA contamination introduced by molecular biology grade water, PCR reagents and DNA extraction kits.

*Alteromonas macleodii* is commonly found in temperate or tropical sea waters [27, 28]. The presence of these Gram-negative bacteria in humans has not been reported. The present study detected high levels of *A. macleodii* reads across the samples, accounting for 0.51–11.35 % of non-host reads. It is suspected that this organism is a contaminant, and its presence in the NGS dataset should be disregarded.

Bacteriophage (phage) infects and replicates within a bacterium and can be present in the study samples through multiple routes. As *Enterobacter* is part of normal gut flora, there is obviously abundant enterobacteria phage in the human body. Assuming that the phage originated from the patients’ gut, the question is how this phage can escape the gut–blood barrier. On the other hand, sequencing of phage genomes is an interesting field of research, with potential uses for phages as antimicrobials and biocontrol agents for food production [29], and the phiX174 bacteriophage was the first DNA-based genome to be sequenced, dating back to 1977 [30]. A previous study [31] reported enterobacteria phage phiX174 sensu lato as a common contaminant in NGS datasets from blood samples.

The presence of hepatitis C virus and dengue virus in all samples is evidence of cross-contamination from one sample to another. One sample was collected from a patient with confirmed hepatitis C virus infection (patient ID# 006); however, this sample was not sequenced due to insufficient amounts of nucleic acids to enter the amplification step. Accordingly, it was presumed that the low abundance (<0.1 %) of hepatitis C virus reads in all samples was the result of contamination during DNA/RNA isolation. As for dengue virus, although it was detected in all samples, its presence with high read counts (2.58 %) in sample ID# 5c indicates a true infection. Dengue virus reads were also present in a relatively higher proportion (0.01 %) in sample ID# 14 compared to the rest of the samples. It is possible that patient ID# 014 had a dengue infection.

Human herpesvirus 4 or Epstein–Barr virus (EBV) is one of the most common viruses in humans. This virus is widespread internationally, and around 95 % of the human population is infected with EBV [32]. Therefore, the presence of low levels (<1 % of non-host reads) of EBV in all samples is not surprising. TTMDV has been found in various body fluids, including saliva and nasopharyngeal aspirates, serum, urine and stool collected from children with acute respiratory disease [33]. The frequent detection of TTMDV in our study is consistent with a previous metagenomic study [34], which reported that TTMDV constituted the second largest viral community after torque teno virus in the plasma of healthy adults.

Although most of the organisms detected in the samples were presumably contaminants, our data illustrate the high sensitivity of the deep sequencing approach to reveal microbial diversity within a sample. Previous research [35] had successfully detected bacterial 16S ribosomal DNA sequences that were similar to DNA sequences of *Riemerella anatipestifer*, *Pseudomonas fluorescens*, *Propionibacterium acnes*, *Microbacterium schleiferi*, *Stenotrophomonas* and *Pseudomonas putida* in healthy human blood using real-time PCR and traditional sequencing on an ABI PRISM platform. These bacterial sequences presumably originated either from experimental reagents, from the skin during phlebotomy or from the blood itself. Nonetheless, the findings of the study raised the possibility that there is a ‘normal’ population of bacterial DNA sequences in blood that has previously been considered sterile. The use of NGS in the present study facilitated the identification of organisms that might well escape cultivation because of their low burden in the blood or simply because they are unculturable. With only 1 % of the microbial life on earth able to be cultured [36, 37], culture-independent methods, such as deep sequencing, are clearly required in order to extend our knowledge of microbial diversity.

In addition to revealing microbial diversity in human blood, this study provided important information with regards to the sensitivity and cost-effectiveness of the deep sequencing approach for fever investigation. We demonstrated that, with an optimum sample such as that from patient ID# 005, approximately 15 million PE reads (~2 Gb of data) are sufficient to facilitate the diagnosis of dengue virus. When the virus load was sufficiently high, de novo assembly permitted the generation of several contigs corresponding to a nearly full-length genome, thus confirming the diagnosis of dengue fever at a cost of AUD $600 for sample processing, library preparation and deep sequencing.

Bioinformatics analyses are an enormous challenge in metagenomic studies because, while a variety of tools are available for analysing sequence data, they require expert users to assemble them into an effective workflow. The high volume of data generated by NGS technology is often responsible for considerable delays in achieving a robust diagnosis. In our experience, bioinformatics analyses of ~2 Gb of raw sequence data using Kraken can be completed in 1 h. This speed far exceeds that of conventional BLAST searches, which may take more than 24 h to complete. To complete the data analysis as quickly as possible, we used the CLC Genomic Workbench workflow on reads unclassified by Kraken to facilitate the identification of infectious agents in undiagnosed cases (test subjects). In order to validate Kraken findings, we performed CLC analysis on the raw reads originating from control subjects in parallel with Kraken analysis. As Kraken reported an excessive amount (hundreds) of organisms in a single sample, the usefulness of Kraken in the present study relates to the rapid screening of pathogens associated with fever. There is a possibility that ambiguous reads may be mapped to multiple taxa in the MiniKraken database, resulting in the detection of organisms that were not actually present in the sample (false-positive). We suggest that, if the Kraken program is going to be used for diagnosis, additional steps are required to filter the true pathogen or pathogen sequences with clinical relevance. Most importantly, the Kraken findings are best interpreted in conjunction with reliable clinical information and the results of other tests are absolutely necessary to inform diagnosis.

We showed in Table 1 that performing more complex analyses does not guarantee reliability of results, and may, indeed, result in failure to detect the true cause of fever (false-negative). The CLC analysis workflow, which involved pre-processing and assembly prior to the BLAST search, failed to detect the true pathogen in 75 % (6/8) of control samples. Thus, it can be argued that the success of the deep sequencing approach is more likely to be determined by the sample condition and the sequencing dataset rather than the analysis pipeline. It is important to collect samples with high pathogen load. Ideally, samples should be snap frozen in liquid nitrogen to preserve the scarce amount of nucleic acids from the pathogen. It is equally important to keep the sample processing steps to a minimum to avoid contamination and prevent further degradation of the nucleic acids. Providing negative controls and running ‘blanks’ in the same sequencing lanes as the actual samples can make the interpretation of the results easier, as the contaminating agents can be immediately identified and ruled out from the NGS dataset. Finally, the choice of NGS platform contributes to the success of pathogen detection. The long reads produced by the 454 platform increase the specificity of pathogen identification by facilitating the discrimination of pathogen reads from hosts or endogenous flora. Although Illumina platforms generate short reads (currently up to 2 × 150 bp for the HiSeq and Genome Analyzer II and 2 × 300 bp for the MiSeq), this platform can generate sequencing data much faster than the 454 platform, thus providing sufficient read depth or number of sequence reads generated per run to detect pathogens with a high degree of sensitivity.

The development of single-molecule sequencing (also known as third-generation sequencing), such as nanopore sequencing or MinION (Oxford Nanopore Technologies), is highly promising; this pocket-sized genome sequencer can generate longer reads (tens of kilobases) at a cost comparable to that of currently available NGS instruments [38]. Another advantage of this portable DNA sequencer over NGS is its ability to perform real-time sequence analysis, which is highly valuable for providing results rapidly. The downside of nanopore sequencing is that the technology currently requires micrograms of DNA/cDNA input. Two recent studies [39, 40] reported disadvantages of MinION sequencing, including higher error rates (10–30 %) and relatively lower throughput (<100,000 reads per cell) compared with NGS.

In conclusion, the deep sequencing approach facilitated the identification of infectious agents associated with AUF as well as other organisms present in human blood. We identified challenges in conducting a deep sequencing approach for routine investigation of fever. Future improvements in sequencing platforms are needed to provide longer reads and enable sequencing from smaller amounts of input material. The development of bioinformatics tools should be directed towards user-friendly options and the means to provide answers in clinically relevant timeframes (e.g. within hours of sample receipt). Recent advancement in sequencing technologies and bioinformatics analyses provides a positive outlook for the application of the deep sequencing approach to facilitate the diagnosis of AUFs.

## Abbreviations

AGRF: Australian Genome Research Facility
ANA: Antinuclear antibody
ALT: Alanine aminotransferase
AST: Aspartate aminotransferase
AUF: Acute undifferentiated fever
BLAST: Basic Local Alignment Search Tool
CMV: Cytomegalovirus
CRP: C-reactive protein
CT: Computed tomography
DNA: Deoxyribonucleic acid
EBV: Epstein–Barr virus
NCBI: National Center for Biotechnology Information
NGS: Next-generation sequencing
PCR: Polymerase chain reaction
RNA: Ribonucleic acid
SFG: Spotted fever group
TTMDV: Torque teno midi virus
WBC: White blood cell
